# Tetracalcium Phosphate Graft for Implant Stabilization: Resonance Frequency and Histomorphometric Analysis in a Sheep Tibia Model

**DOI:** 10.3390/jfb17020069

**Published:** 2026-01-29

**Authors:** Dogac Mevlut Saltan, Nazlı Ayşeşek, Volkan Arısan, Selim Ersanlı

**Affiliations:** 1 Institute of Health Sciences, Department of Oral Implantology, İstanbul University Faculty of Dentistry, İstanbul 34116, Türkiye; dogacsaltan@hotmail.com; 2 Department of Oral Implantology, İstanbul University Faculty of Dentistry, İstanbul 34116, Türkiye; varisan@istanbul.edu.tr (V.A.); selimersanli@istanbul.edu.tr (S.E.)

**Keywords:** guided bone regeneration, bone graft, primary stability

## Abstract

Background: This study aimed to evaluate the effects of tetracalcium phosphate (TTCP) graft material on the stability and osseointegration of dental implants placed in anatomically compromised bone. Materials and Methods: Six healthy sheep were used following ethical approval. Osteotomies were created in the tibial region and divided into three groups: Group A (control, *n* = 12) with standard osteotomy; Group B (*n* = 12) with enlarged and deepened osteotomy; and Group C (*n* = 36), where osteotomy sites were filled with TTCP prior to implant placement. Implant stability was measured using the resonance frequency analysis (RFA), and osseointegration was evaluated histologically by bone-to-implant contact percentage (BIC%). Animals were sacrificed at the 3rd and 6th weeks for histological analysis. Results: Initial RFA values exceeded 42.5 in all groups. Group C demonstrated the highest RFA at Week 6 (79) and significantly higher RFA values at Week 3 compared to other groups, while Group B consistently showed the lowest stability. At Week 3, Group A exhibited the highest BIC% (28.04 ± 5.05%). By Week 6, BIC% increased in all groups, with no significant intergroup differences. Robust ANOVA revealed significant effects of time and group on both RFA and BIC%. Conclusions: TTCP significantly enhanced implant stability and osseointegration in compromised bone, providing improved secondary stability and suggesting its potential clinical benefit in challenging anatomical conditions.

## 1. Introduction

The long-term success of dental implants relies immensely on achieving and maintaining sufficient primary and secondary stability, particularly in cases where bone quantity or quality is compromised [1]. Achieving sufficient primary mechanical anchorage, and thus primary implant stability, is often challenging in post-extraction sockets, atrophic ridges, or poor-quality alveolar bone, frequently requiring grafting or staged procedures [2]. These interventions, while effective, may delay treatment timelines, increase costs, and elevate the risk of complications [3].

Primary implant stability (PIS) is a critical determinant for osseointegration and can be quantified both mechanically and histologically. The implant stability quotient (ISQ), obtained via resonance frequency analysis (RFA), offers a non-invasive method to monitor stability across the healing phase [4]. Additionally, bone–implant contact percentage (BIC%) remains a key histomorphometric parameter reflecting osseointegration [5,6]. PIS and BIC% can be compromised due to several factors, such as poor bone quantity and quality, as well as operator-dependent errors, such as inadequate manipulation during osteotomy. Implants with insufficient PIS are less likely to osseointegrate and carry a higher risk of early-term failure [7].

Various surgical and biomaterial-based strategies have been suggested to enhance PIS in compromised sites such as those with poor bone density or residual extraction sockets [8]. Among these, the use of bone substitutes packed within the osteotomy site has been shown to enhance mechanical interlocking and provide a more favorable environment for early bone regeneration [9,10]. Autogenous bone chips, xenografts, and calcium phosphate-based materials have been used to clog oversized osteotomies or fresh extraction sockets, aiming to increase the frictional contact between the implant and the surrounding bone while promoting new bone formation along the implant threads [11,12]. Such approaches are particularly valuable when implant stability is compromised due to poor bone quality, cortical perforation, or insufficient cortical engagement [13].

Calcium phosphate-based biomaterials have attracted interest due to their biocompatibility, osteoconductivity, and chemical similarity to native bone mineral [14]. Among these materials, TTCP is notable for its adhesive properties and ability to promote mineralization, making it a promising candidate for enhancing implant stability in compromised conditions. TTCP-containing pastes and cements have demonstrated favorable biological interactions, including support for osteoblast activity, extracellular matrix deposition, and mild resorption kinetics [15]. Although hydroxyapatite, β-tricalcium phosphate and octacalcium phosphate are widely utilized, tetracalcium phosphate (TTCP) stands out with its in situ adhesive and hardening properties [16]. Unlike particulate grafts, TTCP can provide immediate mechanical interlocking between the implant and surrounding bone walls, which is particularly advantageous in extraction sockets where primary implant stability is compromised due to the gap between the socket walls and the implant body [17].

The aim of this study was to assess the effect of a TTCP filler graft on the stability and osseointegration of titanium dental implants in a sheep tibia model. The null hypothesis was that the stability and osseointegration changes in titanium implants inserted in the compromised osteotomy sites are not associated with statistical significance when supported by a TTCP filler grafting material.

## 2. Materials and Methods

### 2.1. Sample Size Calculation

The primary outcome was the bone-to-implant contact percentage (BIC%) and a dedicated software was used G*Power v3.1.9 (Heinrich Heine University Düsseldorf, Germany). The statistical power of the study was defined as 1-β (β = probability of Type II error). Based on the bone-to-implant contact percentage (BIC%) values reported in a previous similar study [18], and assuming a significance level of α = 0.05 with 80% power, the effect size (d) was calculated to be 1.978. Accordingly, the sample size was planned as *n* = 6 for each of the two control groups and *n* = 18 for the test group. The number of sites in the test group was intentionally increased to sustain statistical power above 80% in case of unforeseen events like animal death or early implant failure. Considering that 10 implant sites were created per animal, the use of three animals per healing period (3 weeks and 6 weeks) (resulting in a total of six animals) was deemed sufficient to meet the required power level.

### 2.2. Animal Experiment

This study was conducted with the approval of the Animal Ethics Committee of the Mehmet Akif Ersoy Experimental Research and Development Center in İstanbul, Türkiye (Approval No.: 2019/21). A total of six healthy adult male sheep were utilized, in accordance with the institutional guidelines for animal care and use. All animals were fasted for 24 h before the surgical procedures. The tibia was selected as the surgical site to minimize the risks of postoperative infection and early implant failure.

All surgical procedures were performed with general anesthesia under sterile conditions. Sedation was achieved via intramuscular injection of Xylazine (Rompun, Bayer, İstanbul, Türkiye) at a dose of 0.1 mg/kg. Following adequate sedation, an angiocatheter was inserted into the cephalic vein (V. cephalica antebrachii), and anesthesia was induced using 2 mg/kg ketamine hydrochloride (Ketalar^®^, Pfizer, New York, NY, USA). The animals were subsequently intubated, and general anesthesia was maintained with 2–3% isoflurane in 100% oxygen during the surgical procedure.

Animals were positioned in sternal recumbency. The proximal tibia region was shaved and disinfected with povidone–iodine (Batticon^®^, Adeka, Samsun, Türkiye). An incision of approximately 25 cm was made, skin and fascia were incised, respectively, and muscles were dissected. A longitudinal skin incision was made parallel to the tibial axis, followed by blunt dissection of the subcutaneous tissue to expose the bone surface. In the metaphyseal region of each tibia, five standardized bone defects were prepared in a single row using a surgical motor under continuous irrigation with sterile saline. A distance of 10 mm (edge-to-edge) was maintained between each osteotomy site to prevent interaction between the specimens.

Three experimental groups were created to simulate the following groups.

**Figure 1:**
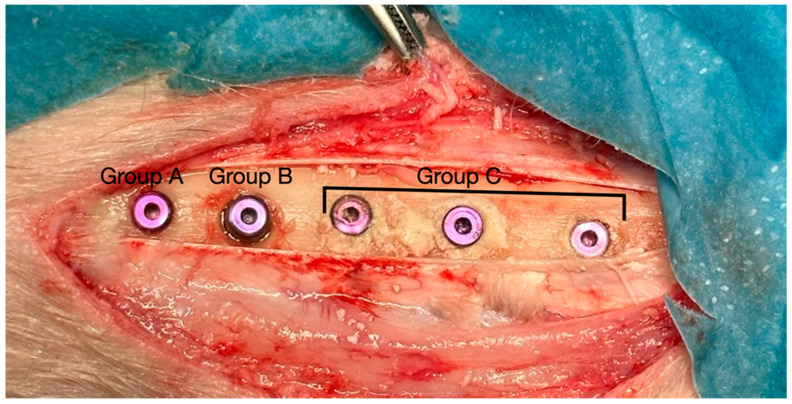
Intraoperative view of the sheep proximal tibia showing the experimental implant sites and group distributions. Surgical procedures were performed bilaterally on both tibias of each of the six animals. Group A (standard osteotomy + implant), Group B (wide osteotomy + implant), and Group C (wide osteotomy + TTCP + implant).

**Figure 2:**
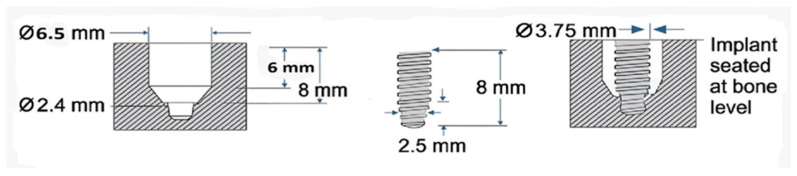
Geometric scheme of the experimental model representing extraction socket and simultaneous implant insertion (immediate implantation); (Group B and C).

Group A (control; *n* = 12) consisted of standard osteotomies prepared according to the manufacturer’s recommended drilling protocol for a 3.75 mm × 8 mm commercially pure titanium dental (Forever Implant, Seoul, Republic of Korea). Group B (*n* = 12) followed the same osteotomy sequence as Group A; however, the osteotomies were enlarged to a diameter of 6.5 mm to simulate an extraction socket. The implants were inserted in a centralized position to simulate immediate implant placement. Group C (*n* = 36) was identical to Group B in terms of osteotomy dimensions; however, the prepared osteotomy cavities were filled with a TTCP graft material (Permed, Çanakkale, Türkiye) prior to implant insertion. Implants were positioned centrally to simulate immediate implant placement with simultaneous grafting (Figure 1 and Figure 2). All implants were commercially available with a sand-blasted and acid-etched surface. TTCP was used as a grafting material only. To minimize potential variation in cortical thickness and cancellous bone quality, the allocation of the A, B and C groups was randomly assigned in the tibia, ensuring balanced and equal distribution within the animal.

Immediately after implant placement, ISQ values were measured using a dedicated RFA device (IS3 ISQ Monitor, Hiossen^®^ Implant, Englewood Cliffs, NJ, USA) and cover screws were inserted into the implants. Flap closure was achieved by sequential repositioning of the muscles, fascia, and skin. Soft tissue closure was performed in two layers: the deep fascia was sutured with absorbable Vicryl 2-0 sutures, and the skin was closed with non-absorbable Prolene 2-0 sutures. The same surgical approach was applied bilaterally to both tibias. Antibiotics (Novosef 1 g, 20 mg/kg, intramuscular; Zentiva, İstanbul, Türkiye) and analgesics (Melox 0.1 mg/kg, intramuscular; Nobel Drug, İstanbul, Türkiye) were administered during postoperative care for 1 week. Sutures were removed 10 days after surgery. To represent early- and late-term healing, three sheep were sacrificed at 3 weeks, and the remaining three sheep were sacrificed at 6 weeks.

At the end of the healing periods, animals were euthanized under high doses of isoflurane. Prior to tissue harvesting, RFA measurements were repeated for all implant sites. Subsequently, the implant-bearing segments were retrieved en bloc and submitted for histological processing to evaluate bone-to-implant contact percentage (BIC%).

### 2.3. Histologic and Histomorphometric Evaluation

Bone segments were fixed in 10% neutral buffered formalin at 4 °C for 15–30 min, then further fixed for 6–12 h. Tissues were trimmed to 2–4 mm in thickness to ensure optimal fixation. Dehydration was carried out over 24–32 h using graded alcohol or glycol methacrylate, depending on sample thickness. Specimens were infiltrated under vacuum with a 1:1 mixture of glycol methacrylate and embedding resin (Technovit^®^ 7200 VLC, Kulzer GmbH., Wehrheim, Germany), then embedded in transparent molds under vacuum to prevent air entrapment. Polymerization was achieved under low-intensity yellow light (4 h) and finalized with high-intensity blue light (450 nm) for 4–10 h, based on sample thickness. The cured blocks were mounted on slides using Technovit^®^ 7210 and prepared using the Exact^®^ micro-grinding system. Sections (~100 µm) were cut and polished with progressively finer abrasives. Diamond-coated discs were used for samples containing metal. Finalized sections were sealed with Technovit^®^ 7200 or paraffin. Histomorphometric evaluations were performed using Bioquant Osteo II software.

Sections were prepared with a non-decalcified histologic slicing system (Exact 300 CL; Exakt Apparatebau, Norderstedt, Germany). The sections were analyzed using a light microscope (Olympus BX60, Tokyo, Japan) to measure the BIC%. All measurements were made using an image analysis software (Olympus Image Analysis System; Olympus Soft Imaging Solutions GmbH, Münster, Germany). The implant surfaces were analyzed in three adjoining microscopic images. The BIC% was measured at a magnification of 40×. The calculation was performed by dividing the length of the attached bone by the length of the complete implant surface (including the whole threads, but the platform surface was excluded).

### 2.4. Statistical Analysis

Data were analyzed using Minitab (Version 14) and Jamovi (Version 2.3.28) software. The Shapiro–Wilk test (W) was applied to assess the normality of data distribution. Robust analysis of variance (Robust-ANOVA) and Bonferroni post hoc tests were used for comparisons that did not show normal distribution. Two-way ANOVA and Bonferroni tests were used for data that comply for a normal data distribution. Quantitative data are presented as mean ± standard deviation (normal data distribution) and median (inter-quartile range (IQR); non-normal data distribution), with a significance level set at *p* < 0.05.

## 3. Results

All osteotomies were completed successfully, and despite the oversized osteotomy executed in Group B, the corresponding implants achieved stability. Post-op healing was uneventful, and no complications were observed through the healing period.

### 3.1. RFA Values

Initial RFA values were above 42.5 in all groups and reached a maximum of 79 (Group C) at the end of 6 weeks. In Groups A and B, RFA values showed an initial decrease followed by an increase during the 3- and 6-week healing periods. However, in Group C, no noticeable decrease in RFA was observed. Throughout the healing period, the lowest RFA values were recorded in Group B. By the 3rd week of healing, Group C demonstrated significantly higher RFA values.

Distribution of RFA values was not normal (*p* = 0.022). Robust ANOVA revealed that RFA values varied significantly over time (F = 35.9, *p* < 0.001) and between groups (F = 306.0, *p* < 0.001), with a significant time × group interaction (F = 444.4, *p* < 0.001).

At baseline, the lowest RFA readings were observed in Group B (median = 48 ISQ, IQR: 47, 25–49), whereas the highest values were recorded in Group A (median = 73 ISQ, IQR: 69–73, 75). Pairwise comparisons showed that the RFA values in Group B were significantly lower than those in both Group A and Group C (median = 70 ISQ, IQR: 67–75, 75; Robust ANOVA, *p* < 0.001).

At the third week of healing, RFA values differed significantly among all groups (Robust ANOVA, *p* < 0.001). Group C exhibited significantly higher values (median = 72 ISQ, IQR: 69–73, 25) compared with both Group B (median = 42.5 ISQ, IQR: 41–45, 25) and Group A (median = 66.5 ISQ, IQR: 64, 25–68, 25).

**Figure 3:**
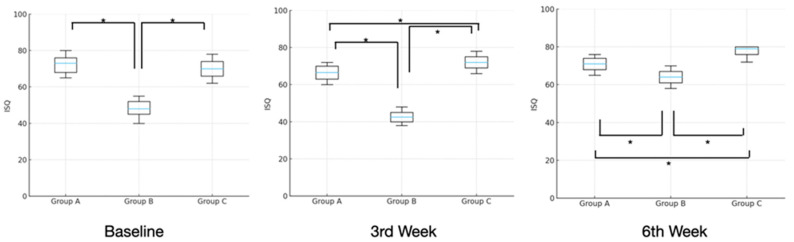
Box-and-whisker plots illustrating the distribution of RFA values (median and quartiles) across the examination periods in the groups. (* *p* < 0.05).

By the sixth week, Group C (median = 79 ISQ, IQR: 77–80) maintained significantly higher RFA values than Group A (median = 71 ISQ, IQR: 65–74, 50) and Group B (median = 64 ISQ, IQR: 62, 50–66, 75; Robust ANOVA, *p* < 0.001) (Table 1 and Figure 3).

### 3.2. Bone Histomorphometry

Normal distribution of BIC% data was confirmed (*p* = 0.082). Robust ANOVA revealed that BIC% values significantly varied in time (F = 372.544, *p* < 0.001, η^2^ = 0.873) and groups (F = 10.306, *p* < 0.001, η^2^ = 0.276). A significant Group × Time interaction was also detected (F = 4.975, *p* = 0.010, η^2^ = 0.156), indicating that the change in BIC% over the healing period differed among the groups.

At the third week, there was a significant difference in BIC% among the groups, with the highest BIC% observed in Group A (28.04 ± 5.05%) (*p* < 0.001). By the sixth week, BIC% values had increased in all groups, and the differences between them were no longer statistically significant.

**Figure 4:**
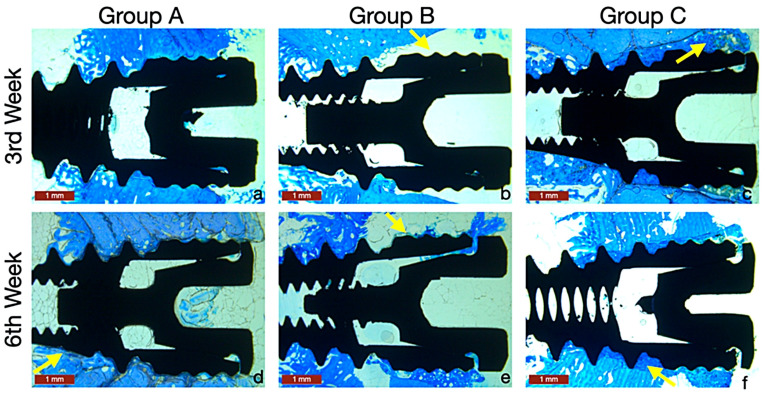
Representative histologic images of toluidine blue-stained sections in the groups after 3 and 6 weeks of healing. (**a**–**c**) At 3rd week, limited new bone formation was observed around the implant threads. Group A exhibited closer bone contact with the implant surface, whereas Group B showed wide gaps (arrow) between the bone and the implant and Group C displayed areas of newly forming bone along the coronal region (arrow). (**d**–**f**) At 6th week, all groups showed increased bone deposition compared with the 3rd week. Continuous bone contact along the implant surface was evident in Groups A and C (arrows), while Group B still demonstrated incomplete bone fill at the coronal region. (Toluidine blue staining; original magnification ×40).

**Figure 5:**
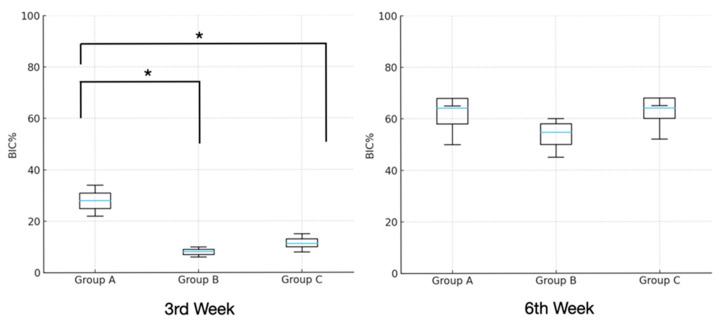
Box-and-whisker plots showing median and quartile values for the BIC% values. (* *p* < 0.05).

Overall, Group A (46.11 ± 21.48%) showed the greatest mean BIC% across the study period, followed by Group C (37.64 ± 27.62%) and Group B (31.48 ± 25.28%). (Table 2), (Figure 4 and Figure 5).

## 4. Discussion

This study aimed to evaluate the effects of a tetracalcium phosphate (TTCP)-based graft material on implant stability and bone-to-implant contact (BIC%) in a sheep tibia model, simulating clinical situations with compromised recipient bone or osteotomy site. Initial low RFA and BIC%’s measured at the baseline and earlier stages of healing of compromised osteotomy sites appear to have diminished the differences rapidly via the support of the TTCP at the end of the 6 weeks.

Tetracalcium phosphate (TTCP) is biocompatible and osteoconductive and has demonstrated favorable outcomes in various preclinical models, including rats, rabbits, dogs, and sheep [19]. Composed of TTCP, phosphoserine, and water, the graft used in the present study sets with minimal exothermic reaction, reducing the risk of thermal tissue damage during application [1,20]. Once applied, it hardens in situ and adheres firmly to both bone and titanium surfaces, offering an initial mechanical interlock while also facilitating cellular adhesion and bone matrix mineralization [21]. The resulting microporous, biologically favorable scaffold supports space maintenance, soft tissue integration, and biocompatibility, all of which are critical in cases where native bone is compromised due to inappropriate osteotomy and/or anatomy [22]. In clinical scenarios such as extraction sockets or inappropriately widened osteotomies, where conventional implant placement may not achieve optimal primary stability, TTCP presents a promising solution by serving both as a mechanical stabilizer and a biologically active matrix [23].In this study, the inclusion of both wide osteotomies with TTCP and standard osteotomies without grafting allowed us to assess whether TTCP merely compensates for mechanical deficiency or provides superior outcomes.

At baseline, the implants that were placed in Group A (standard osteotomy; median RFA: 73 ISQ) and Group C (wide osteotomy + TTCP; median RFA: 70 ISQ) exceeded the clinically accepted threshold for primary stability (RFA > 60 ISQ), as previously defined by Sennerby and Meredith (2008) [24]. In contrast, Group B (wide osteotomy without graft) exhibited significantly lower stability (median: 48 ISQ), highlighting the negative biomechanical consequences of oversized osteotomies [25]. The ability of TTCP-augmented implants in Group C to nearly match the RFA values of standard osteotomy despite compromised initial bone contact suggests that TTCP plays a crucial role in restoring primary mechanical stability, potentially via both adhesive and cohesive mechanisms.

At the 3rd week, Group C continued to outperform the other groups, achieving the highest RFA (median: 72 ISQ), significantly greater than both Group A (66.5 ISQ) and Group B (42.5 ISQ). This is noteworthy considering that week 3 typically represents the “stability dip” during bone remodeling, where mechanical stability is expected to decline due to transient weakening of the bone-implant interface [26,27]. The results were similar to the study of Vandeweghe et al. (2011), where surface-modified implants showed superior RFA maintenance through early healing [25]. Moreover, similar to the results observed with hydrofluoric acid-etched implants [24], TTCP may provide a favorable surface environment that sustains mechanical integration during this biologically vulnerable period.

By the 6th week, Group C demonstrated a further increase in RFA (median: 79 ISQ), surpassing both Group A (71 ISQ) and Group B (64 ISQ). This progressive increase is consistent with the secondary stability phase, where new bone formation reinforces the interface. The pattern resembles the findings of Tadi et al. (2014), in which initial implant stability was recorded with a mean ISQ of 55 and an insertion torque of 36.50 N/cm, and RFA values in immediate implants gradually increased over a 6-week period, supporting the notion that bioactive fillers such as TTCP may enhance both early and late mechanical stability [28].

Histologically, although Group A exhibited the highest BIC% at 3 weeks (28.04 ± 5.05%), Group C showed lower initial bone contact (11.29 ± 2.20%), likely due to the interposition of the graft material. This initial delay in osseointegration is a well-known phenomenon with calcium phosphate materials, as seen in prior animal studies (Shin et al., 2015) [19]. However, by week 6, Group C’s BIC% increased markedly, nearly equaling that of Group A, while Group B remained consistently low. This delayed but robust improvement aligns with findings from Piccinini et al. (2013), who similarly observed thicker bone trabeculae, persistent HA/TTCP granules, and a 64% bone graft contact at 17 weeks, demonstrating TTCP’s sustained osteoconductive potential [29]. In Cochran’s study, the mineral-organic adhesive demonstrated markedly superior early bone-to-implant contact (BIC), with a combined mineralized material contact of 80% at 10 days, compared with 7% in the negative control and 6% in the bovine bone-grafted sites (*p* < 0001). By 4 months, the adhesive had largely resorbed and was replaced by newly formed bone, resulting in a BIC of 44%, which remained significantly higher than that of the bovine graft group (*p* = 0.0335) and numerically above the negative control (35%). These findings indicate that the mineral-organic adhesive supports early crestal stability and promotes a statistically significant and sustained increase in BIC as healing progresses [15,16,17,18,19,20,21,22,23,24,25,26,27,28,29,30].

Taken together, the divergence between RFA and BIC% values in the early phase highlights that implant stability is influenced not only by bone contact but also by the graft’s mechanical and adhesive properties. As emphasized by Aparicio et al. (2006), RFA should be interpreted within a broader biological and mechanical context [31]. In this respect, TTCP’s dual contribution (providing initial mechanical support and later biological integration) makes it a promising adjunct in clinical scenarios where native bone is insufficient or immediate stability is compromised. Taken together with the previously reported reliable long-term biodynamic properties of TTCP, the method of application employed in the present study (Group C) may represent a safe and biocompatible approach for immediate dental implant placement [8,10,18].

This animal experiment was designed to compare immediate implantation against conventional implant placement under controlled conditions. Nevertheless, the anatomical site used in this study and its healing characteristics may not fully reflect the complex biological environment of the human oral cavity. Moreover, the biological behavior of the TTCP graft material may differ in human clinical settings. Therefore, the results of this study should be interpreted with caution when considering their direct clinical translation [21].

The present evidence suggests that TTCP grafts in compromised implant recipient bone (wide osteotomy defects) improve primary and secondary implant stability. This has potential utility in clinical scenarios for immediate implantation. However, reduced early BIC% suggests that healing protocols might require adjustment (such as delayed loading or extended healing phases) when employing TTCP graft in combination with immediate implantation techniques.

## 5. Conclusions

Within the limitations of this preclinical investigation, the present study provides compelling evidence that tetracalcium phosphate (TTCP) significantly enhances both mechanical and biological aspects of implant stability in anatomically compromised conditions. TTCP demonstrated comparable primary stability to standard osteotomy at the time of placement and notably superior secondary stability by Week 6, as reflected by higher RFA values and increased bone-to-implant contact (BIC%) ratios. The progressive replacement of the biomaterial interface with newly formed bone, accompanied by a reduction in soft tissue interference, underscores TTCP’s osteoconductive and bioactive potential. These outcomes suggest that TTCP may serve as a clinically valuable adjunct in cases where achieving conventional primary stability is challenging, such as immediate implantation or in sites with intrinsic osteotomy errors. While these findings are promising, future long-term studies in human models are essential to validate the translational applicability of the presently investigated TTCP-supported dental implant model.

## Figures and Tables

**Table 1:** Analysis of RFA values (ISQ) using median-based Robust ANOVA. (Groups sharing the same letter (^a–c^) are not significantly different. Interactions sharing the same letter (^A–F^) are not significantly different).

Time Point	Group A	Group B	Group C	Overall Median (Min–Max)	Test Statistic	*p* *
**Baseline**	73 (68–81) ^A^	48 (47–52) ^D^	70 (64–84) ^A^	69 (47–84) ^a^	56.04	<0.001
**Week 3**	66.5 (62–69) ^BC^	42.5 (41–46) ^E^	72 (67–74) ^A^	69 (41–74) ^b^	49.47	<0.001
**Week 6**	71 (62–76) ^AB^	64 (61–69) ^C^	79 (72–82) ^F^	75 (61–82) ^c^	80.45	<0.001
Total	69 (62–81)^a^	48 (41–69) ^b^	72.5 (64–84)^c^	70 (41–84)		

* Robust ANOVA using medians for the comparison. Values are presented as median (minimum–maximum). Superscript lowercase letters (^a–c^): groups sharing the same letter are NOT significantly different. Superscript uppercase letters (^A–F^): interaction cells (group × time combinations) sharing the same letter are NOT significantly different. Global robust ANOVA results: Group effect test statistic = 306.00, *p* < 0.001; Time effect test statistic = 35.90, *p* < 0.001; Group × time interaction test statistic = 444.40, *p* < 0.001.

**Table 2:** Analysis of BIC% values using Two-way ANOVA. Values are presented as mean ± standard deviation (SD). Superscript lowercase letters (^a,b^): overall group means sharing the same letter are NOT significantly different. Superscript uppercase letters (^A–C^): group × time interaction cells sharing the same letter are NOT significantly different. Model performance: R^2^ = 91.49%, Adjusted R^2^ = 90.71%.

Time Point	Group A (Mean ± SD)	Group B (Mean ± SD)	Group C (Mean ± SD)	Overall Mean ± SD	Factor	F	*p*	Partial η^2^
**Week 3**	28.04 ± 5.05 ^B^	8.24 ± 1.54 ^C^	11.29 ± 2.20 ^C^	14.03 ± 7.74	Group	10.306	<0.001	0.276
**Week 6**	64.17 ± 14.38 ^A^	54.72 ± 10.34 ^A^	64.00 ± 9.77 ^A^	62.18 ± 11.15	Time	372.544	<0.001	0.873
Total	46.11 ± 21.48 ^a^	31.48 ± 25.28 ^b^	37.64 ± 27.62 ^b^	38.10 ± 26.07	Group × Time	4.975	0.010	0.156

## Data Availability

The original contributions presented in this study are included in the article. Further inquiries can be directed to the corresponding author.
